# Autophagy and Its Role in Protein Secretion: Implications for Cancer Therapy

**DOI:** 10.1155/2018/4231591

**Published:** 2018-12-06

**Authors:** Israel Cotzomi-Ortega, Patricia Aguilar-Alonso, Julio Reyes-Leyva, Paola Maycotte

**Affiliations:** ^1^Facultad de Ciencias Químicas, Benemérita Universidad Autónoma de Puebla, Ciudad Universitaria, Puebla 72570, Mexico; ^2^Centro de Investigación Biomédica de Oriente, Instituto Mexicano del Seguro Social, Km 4.5 Carretera Atlixco-Metepec HGZ5, Puebla 74360, Mexico; ^3^CONACYT-Centro de Investigación Biomédica de Oriente, Instituto Mexicano del Seguro Social, Km 4.5 Carretera Atlixco-Metepec HGZ5, Puebla 74360, Mexico

## Abstract

Autophagy is a protein and organelle degradation pathway important for the maintenance of cytoplasmic homeostasis and for providing nutrients for survival in response to stress conditions. Recently, autophagy has been shown to be important for the secretion of diverse proteins involved in inflammation, intercellular signaling, and cancer progression. The role of autophagy in cancer depends on the stage of tumorigenesis, serving a tumor-suppressor role before transformation and a tumor-survival function once a tumor is established. We review recent evidence demonstrating the complexity of autophagy regulation during cancer, considering the interaction of autophagy with protein secretion pathways. Autophagy manipulation during cancer treatment is likely to affect protein secretion andinter-cellular signaling either to the neighboring cancer cells or to the antitumoral immune response. This will be an important consideration during cancer therapy since several clinical trials are trying to manipulate autophagy in combination with chemotherapy for the treatment of diverse types of cancers.

## 1. Introduction

Macroautophagy (referred herein as autophagy) is an evolutionary conserved catabolic and quality control process which involves the formation of double-membraned vesicles known as autophagosomes that engulf cytoplasmic proteins and organelles for their degradation in the lysosome [1]. Basal levels of autophagy are normally low but are induced upon exposure to starvation or diverse types of stress, indicating an important role for autophagy during metabolic homeostasis [2]. The housekeeping role of basal autophagy is evidenced by the accumulation of autophagy substrates like damaged proteins and organelles after genetic ablation of the process in a diversity of models [2]. Also, upon stimulation of stress-induced autophagy, the cells use their breakdown products for obtaining energy and to generate metabolic precursors for cell survival [3].

The importance of autophagy in health and disease was acknowledged by the award of the 2016 Nobel Prize in Physiology or Medicine to Dr. Yoshimori Ohsumi for the discovery of the Atg proteins, the proteins regulating the autophagic process [4]. Dr. Ohsumi's discovery led to the investigation of autophagy in different research areas and to a deeper understanding of the process and its regulators which has led to studies that suggest the possibility of therapeutically targeting autophagy for the treatment of diverse diseases.

The development of mutant mice deficient in ATG proteins demonstrated that autophagy is essential for survival during the neonatal stage of development in mammals [5]. The first mutant mice generated with knockout of an *Atg* gene were the *Atg5* knockout mice [6]. These mice showed reduced amino acid levels in tissues and plasma, died neonatally with a lack of obvious anatomical abnormalities at birth, and presented a suckling defect. Since autophagy is massively induced after birth in response to starvation caused by the termination of the transplacental nutrient supply, the absence of autophagy together with the suckling defect of the mutant mice has been proposed to be responsible for the inability to restore nutrient supply and the observed neonatal lethality [6]. Although artificial milk feeding partially extended the survival of *Atg5*-null neonates, *Atg5*-null mice also presented defects in the clearance of apoptotic corpses and in the development of the heart and lung. More recently, it has been demonstrated that neuronal dysfunction in *Atg5* knockout mice is the main cause of neonatal lethality, since re-expression of *Atg5* in the brain was sufficient to avoid lethality in this model [7]. These findings underscore the importance of the autophagic pathway for proper organismal development and as a major generator of amino acids under starvation condition to maintain cellular and organismal viability.

A similar phenotype has been observed in *Atg3*, *Atg7*, *Atg12*, and *Atg16L1* knockout and *Ulk1/2* double-knockout mice [5, 8]. Yet, *beclin1*, *Ambra1*, and *Rb1cc1/FIP200* knockout mice are unable to produce homozygous offspring due to early embryonic lethality, suggesting that these genes have other important functions during development in addition to their participation in autophagy [5, 7].

To investigate the role of autophagy in a fully developed organism, adult mice subjected to conditional whole-body deletion of *Atg7* have been generated [9]. These mice developed tissue damage, including liver enlargement, decreased number of large pyramidal neurons and Purkinje cells, degenerative changes in muscle, and vacuolization in the pancreas. *Atg7* conditional knockout mice succumbed either to *Streptococcus* infection shortly after *Atg7* deletion or to neurodegeneration 2 to 3 months later [9]. Importantly, after *Atg7* inactivation, mice failed to survive fasting for 24 hours. Thus, adult mice are less autophagy-dependent than neonates since they can survive longer in the absence of autophagy. However, the autophagic process is necessary for adult tissue maintenance, especially neuronal maintenance in fully developed organisms and essential for organismal survival during fasting [2, 9].

The fact that the autophagic process has a central role in adult neuronal maintenance and in the removal of protein inclusions within neurons (like the ones occurring in Alzheimer's, Hungtington's and Parkinson's diseases) as well as in the removal of damaged mitochondria (like the ones accumulating in some familiar forms of Parkinson's disease [10]) has led to numerous clinical trials trying to induce autophagy by different means in neurodegenerative diseases [11]. Thus, it seems that diseases most likely to be treated soon with autophagy modulators in the clinic involve neurodegenerative diseases as well as cancer [11]. Importantly, as we will discuss later, autophagy has also been shown to be important for extracellular plaque formation and lateral transmission of the disease during neurodegeneration, underscoring the importance of considering every consequence of the manipulation of autophagy in the clinic.

Therapeutic targeting of autophagy in cancer is not straightforward, and evidence suggests that a careful selection of patients based on the characteristics of their tumor needs to be made when trying to manipulate autophagy for cancer therapy. However, most clinical trials trying to modulate autophagy for the treatment of cancer are using diverse drugs with the purpose of inhibiting autophagy [11]. Controversies in the field of autophagy manipulation for cancer treatment arise from the fact that autophagy has been implicated in several steps of the tumorigenic process where both tumor-promoting and tumor-suppressor functions for autophagy have been described [12]. More recently, autophagy has also been related to the extracellular release of cytoplasmic components, including proteins and particulate substrates in a process termed secretory autophagy [13], adding more complexity to the multiple roles of autophagy in cell homeostasis, signaling, and its alterations in disease. Here, we review recent evidence relating the autophagic machinery to cellular secretion with a special focus on carcinogenesis, cancer progression, and possible opportunities to improve cancer treatment.

## 2. The Autophagic Pathway

**Figure 1:**
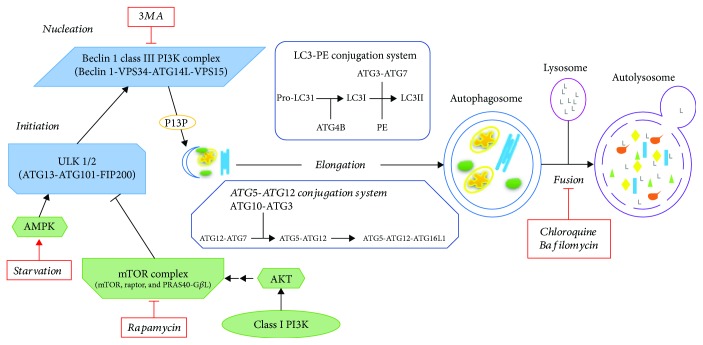
A general overview of the autophagic pathway and its regulators. In mammals, the ULK1/2 kinase complex regulates autophagosome initiation. ULK1/2 is regulated by nutrient sensing or stress signaling by mTOR complex 1, which inhibits autophagy in the presence of amino acids or insulin/PI3K/AKT signaling. ULK1/2 is also regulated by AMPK, which is activated by high AMP/low ATP levels. Activated ULK1/2 then phosphorylates and activates components of the class III PI3K nucleation complex responsible for the formation of PI3P and for the recruitment of PI3P-binding proteins. Vesicle elongation is mediated by two ubiquitin-like protein conjugation systems: ATG5-ATG12 and LC3-PE. Once the autophagosome is formed, it fuses with the lysosomes and their contents are degraded. The figure shows pharmacological regulators of autophagy mentioned in the text (ATG: autophagy related; mTOR: mechanistic target of rapamycin; PI3K: phosphatidylinositol 3-kinase; PE: phosphatidylethanolamine; PI3P: phosphatidylinositol 3-phosphate; AMPK: AMP-activated protein kinase; 3MA: 3-methyl adenine).

The autophagic process is regulated by a set of evolutionary conserved genes termed *ATG* or “autophagy-related” genes, and it comprises the following steps: initiation of the formation of the autophagosome, nucleation, expansion, and elongation of the autophagosomal membrane, closure, and fusion with the lysosome terminating in the degradation of intravesicular products (Figure 1). For an extensive review of this process, the readers are referred to excellent published reviews [1, 14, 15].

Briefly, the Atg1/ULK1/2 kinase complex (in mammals, formed by ULK1/2, ATG13, FIP200, and ATG101) regulates the induction of autophagosome formation. During the first step of autophagy initiation, the ULK1 complex forms punctate structures in proximity to the ER (endoplasmic reticulum), where the nucleation complex is formed. Activated ULK1/2 then phosphorylates components of the class III PI3K (phosphatidylinositol 3-kinase) nucleation complex. This complex consists of a class III PI3K (VPS34), beclin 1, VPS15, and ATG14L. This kinase complex is responsible for the production of the phospholipid phosphatidylinositol 3-phosphate (PI3P) at the site of autophagosome formation that serves as a signaling molecule for the recruitment of PI3P-binding proteins [16]. Vesicle elongation is mediated by two ubiquitin-like protein conjugation systems: ATG5-ATG12 and ATG8/LC3-PE. Both systems are necessary for autophagosome membrane expansion and consist of the following proteins: ATG12 and ATG8/LC3 (ubiquitin like proteins), ATG7 (E1-like enzyme), ATG10 and ATG3 (E2-like enzymes), ATG5 and ATG16 (modified targets), and ATG4 (protease). The ATG5-ATG12 system along with ATG16 functions in part to determine the subcellular localization of ATG8/LC3-PE conjugation. ATG8/LC3 is conjugated to the lipid phosphatidylethanolamine (PE) to form LC3II which is recruited to the autophagosomal membrane and is currently the most widely used assay to evaluate autophagy [15, 17]. LC3II also recognizes adaptor proteins like p62/SQSTM1 which binds ubiquitinated proteins and transports them to the autophagosome. Ultimately, autophagosomes travel along microtubules, pushed by dynein, and fuse with the lysosome and their contents are degraded. Fusion requires ESCRT (endosomal sorting complexes required for transport), SNAREs (STX17), VPS family proteins, and RAB7 [18]. Impaired lysosome function prevents complete autophagic flux. Hence, lysosomotropic agents like chloroquine or hydroxychloroquine, which impair autophagosome degradation and autophagic flux by increasing the pH of the lysosome, are used experimentally and in the clinic in several clinical trials to inhibit autophagy [11, 18].

Autophagy is regulated in response to nutrient availability as well as other cellular stress signals. A master regulator of autophagy in response to nutrient availability is the mTOR (mechanistic target of rapamycin) complex 1 (mTORC1), a serine/threonine protein kinase responsible for regulating cell growth and metabolism. In the presence of amino acids, mTORC1 is active and inhibits autophagy by phosphorylating ULK1, as well as ATG13, at multiple residues [16]. Upon amino acid deprivation, mTORC1 activation on the lysosomal surface is disrupted and both ULK1 and ATG13 are dephosphorylated, resulting in ULK1 activation and autophagy induction [16]. Another important regulator of autophagy is AMPK (AMP-activated protein kinase) which is activated by low ATP levels or an increase in the AMP : ATP ratio. AMPK can inactivate mTORC1 through its phosphorylation and can also directly phosphorylate and activate ULK1 at multiple residues inducing autophagy. Other regulators of the ULK1 complex include GSK3-TIP60, AKT, Cul3-KLHL20, and NEDD4L [16].

## 3. Autophagy and Cancer

Research on autophagy in the cancer biology field has led to a general consensus in which the role of autophagy in cancer is dependent on the stage of tumorigenesis [12]. In general, before the appearance of a tumor, autophagy serves a tumor suppressor function in normal cells, eliminating damaged organelles and protein aggregates which could promote genomic instability and tumorigenesis. On the other hand, once a tumor is established, autophagy serves a cell survival function in cancer cells that helps them survive hypoxia, metabolic stress, and anoikis [12]. So, the homeostatic function of baseline autophagy occurring in normal cells ensures continuous removal of superfluous, ectopic, or damaged (and potentially dangerous) entities, including organelles or proteins, operating as a quality control system that maintains cellular fitness [12]. Additionally, autophagic flux can be upregulated in response to stressful stimuli like nutritional, metabolic, oxidative, pathogenic, genotoxic, or proteotoxic stress [12]. This stimulus-induced autophagy serves a cytoprotective function by helping the cells adapt to stress and allowing them to survive.

In agreement with the housekeeping role for autophagy, cancer was the first disease that was linked to a deficiency in the autophagic pathway with the proposal that *Beclin1* functions as a tumor suppressor gene, since it was found to be monoallelically deleted in a high percentage of ovarian, breast, and prostate cancers [10, 19]. Although this proposal was recently challenged and the tumor suppressive functions of Beclin1 remain controversial [20], diverse mouse models with defects in the autophagy machinery caused by whole-body or tissue-specific, heterozygous, or homozygous knockout of *Atg* genes show increased incidence of some malignancies or increased susceptibilities to carcinogens [10, 12]. So, heterozygous deletion of *beclin1* has been associated with enhanced susceptibility to breast, ovarian, and prostate cancer in humans and increased spontaneous malignancies in mice [21]; *Atg4C* knockout mice have been shown to be more prone to develop chemically induced fibrosarcomas [22]; mosaic deletion of *Atg5* in mice induced benign tumor development in the liver [23]; and tissue-specific *Atg5* or *Atg7* knockout increased the appearance of lung carcinomas driven by KRAS^G12D^ or BRAF^V600E^ [24, 25], as well as KRAS^G12D^-driven premalignant pancreatic lesions [26, 27]. Interestingly, mice with an *Atg7* conditional knockout in the liver developed multiple tumors in this tissue and this phenotype was reversed by *p62* knockout, indicating that p62 accumulation due to autophagy suppression contributes to tumor formation [23].

Thus, before the appearance of a malignant lesion, autophagy serves a tumor-suppressive function. The mechanism proposed involves the degradation of damaged mitochondria that could otherwise induce oxidative stress, DNA damage, and genomic instability. These elements of chronic tissue damage could also provoke an inflammatory response that could further promote tumor growth [28]. In this regard, p62/SQSTM1, one of the best characterized substrates of selective autophagy which interacts with LC3 on the isolation membrane as well as with ubiquitinated proteins, has been shown to play a role in the induction of tumorigenesis. p62 can function as a signaling hub through its interacting proteins. Among these, it can activate the TRAF6-Nf*κ*B pathway, facilitate aggregation of caspase-8, bind Keap1, a Cullin3-type ubiquitin ligase for Nrf2, and facilitate the formation of intracellular inclusion bodies [29–31]. Thus, excess accumulation of p62 due to defective autophagy leads to hyperactivation of these signaling pathways which could further contribute to protumorigenic signaling.

On the other hand, once a tumor is formed, there is ample evidence showing that tumor cells need elevated levels of autophagy to survive the stressors found within a tumor and along the metastatic process [32]. Indeed, autophagy has been shown to promote cancer cell survival under hypoxia [33, 34], nutrient deprivation [35], and anoikis [36], indicating the importance of this process for the survival of a tumor cell to the stressors to which it is exposed and suggesting a potential use for cancer therapy in combination with autophagy inhibitors. Autophagy has also been shown to be a cell survival pathway activated during chemotherapy, radiotherapy, and targeted therapies [37], suggesting promising results of clinical trials using the autophagy inhibitors chloroquine or hydroxychloroquine in combination with other therapies in different types of cancers [11]. Also, autophagy has been implicated in the development of resistance to therapy [1, 38, 39], further supporting the use of pharmacological inhibitors of autophagy in combination with traditional chemotherapy or in patients that recur. This last evidence is also in agreement with the suggestion that autophagy is an important process for the maintenance of cancer stem cells [40–43]. Nevertheless, sensitization to therapy by autophagy inhibitors might be cell type- or treatment-dependent [44, 45] and could even show antagonistic effects with chemotherapy depending on the cell type [45]. In contrast to the previous evidence that suggests a potential use for autophagy inhibition in cancer therapy, it has also been shown that autophagy inhibition in cancer cells treated with radiation [46] or immunogenic chemotherapies [47] could impair the therapy-induced antitumoral immune response. Also, there is evidence in which autophagy inhibition by itself promoted epithelial-to-mesenchymal transition in cancer cells [48]. Thus, it remains unclear if autophagy should be targeted during cancer therapy in every cancer type or what therapies should it be used in combination with.

Regarding the type of cancer cell where autophagy should be targeted, it has been shown that cancer cells with certain oncogenic backgrounds might be particularly sensitive to the inhibition of autophagy, even under nutrient-rich conditions. So, cells with activating mutations in the MAPK pathway have been proposed to be “addicted” to autophagy since they show high levels of autophagy under basal, nutrient-rich conditions and are dependent on this pathway for survival [49, 50]. So, inhibition of autophagy for cancer therapy seems to be promising for the treatment of tumors with activating mutations in KRAS or its downstream targets as BRAF like lung [25, 51], pancreas [52], brain tumors [53], or melanoma [54].

Importantly, some of the autophagy-mediated effects observed during cancer therapy seem to involve either the activation or the modulation of the antitumoral immune response [24, 55, 56]. Moreover, some of the protumorigenic effects of autophagy seem to require the release of autophagy-regulated secreted factors which could act in an autocrine or paracrine manner in cancer cells [40, 57]. Thus, a precise understanding of the secreted factors regulated by autophagy will provide important knowledge on the effects of autophagy on tumor cells as well as on the regulation of the tumor microenvironment by autophagy-competent or autophagy-deficient tumor cells.

## 4. Conventional and Unconventional Protein Secretion Pathways

Cell secretion is a fundamental physiological process that delivers soluble proteins and cargoes to the extracellular space. The need to expel substances from the cell serves distinct purposes including cellular growth, homeostasis, cytokinesis, defense, hormonal release, and neurotransmission [58]. In eukaryotes, classical secretion, also known as the conventional secretion pathway, involves release or exocytosis of storage vesicles or secretory granules into the extracellular space [58]. During this process, newly synthesized proteins are translocated into the lumen of the endoplasmic reticulum (ER). Proteins secreted by classical secretion contain in their sequence a characteristic peptide with one or more positively charged amino acids in their amino terminal end followed by 6–12 hydrophobic residues [59]. The signal sequence initiates the transport of the growing polypeptide across the ER membrane into the ER lumen. Usually, classically secreted proteins are synthesized as protein precursors and the N-terminal signal peptide sequence is cleaved from the protein when the polypeptide chain is growing in the ribosome [59]. Proteins are then oligomerized and packed into carrier vesicles that exit the ER at specialized regions. The vesicles assemble into vesiculotubular structure intermediates known as the ER-to-Golgi intermediate compartments that sort proteins for further anterograde flow to the Golgi complex. In the Golgi, proteins are glycosylated to ensure proper protein structure and increased stability and to allow interactions with target proteins. In the trans-Golgi network, secretory proteins are sorted into secretory vesicles that deliver their content to the plasma membrane to result in secretion [60]. Importantly, integral plasma membrane proteins are delivered and integrated to the plasma membrane through membrane fusion by the same trafficking route [58].

Secretory vesicles and secretory granules are distinct vesicular carriers employed in constitutive and regulated secretion, respectively. While constitutive secretion is constantly undergoing in every eukaryotic cell, regulated secretion is additionally present in special types of animal cells like endocrine and exocrine cells and neurons and is exclusively triggered by extracellular stimuli [58]. Examples of regulated secretion include insulin secretion from endocrine pancreatic *β*-cells, secretion of zymogen from exocrine pancreatic cells to digest food, secretion of growth hormone from cells of the pituitary gland, and release of neurotransmitters at the synapse [58]. While many secreted proteins have been identified to be released by the conventional route, many other soluble proteins that are secreted into the extracellular space lack a typical signal peptide and are secreted without entering the conventional ER-to-Golgi pathway of protein secretion.

## 5. Autophagy and Unconventional Protein Secretion

**Figure 2:**
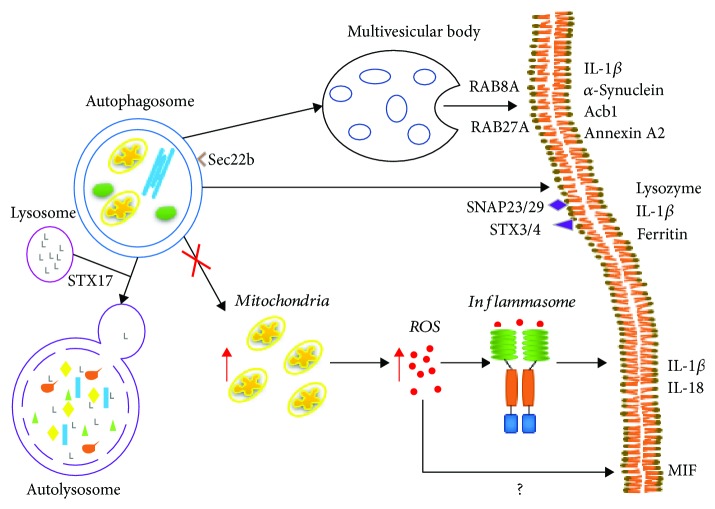
Overview of the different roles of autophagy in protein secretion. Three possible mechanisms of autophagy-mediated secretion have been described. In the first one, the autophagosome interacts with components of the endosomal-lysosomal system, including the multivesicular body. These interactions are mediated by Sec22b, Rab8A, and Rab27A proteins for the release of *α*-synuclein and annexin A2 [69, 70, 86], and only Rab8A has been characterized for the release of IL-1*β* [64]. It should be noted that the secretion of IL-1*β* and other proteins, like ferritin, can also be carried out by direct fusion of the autophagosome to the plasma membrane, mediated by Sec22b and SNAP23/29 and STX3/4 [66], and that the secretion of IL-1*β* is independent of proteins involved in the fusion of the autophagosome with the lysosome such as STX17 [66] probably suggesting a mechanism in which secretory autophagosomes are spared from degradation and instead are directed to the multivesicular body or the plasma membrane. On the other hand, the inhibition of autophagy prevents the degradation of damaged organelles such as the mitochondria, inducing an increase in ROS involved in the secretion of MIF, through an unknown mechanism [80]. A ROS-dependent mechanism induced by decreased mitophagy has been described for other proteins such as IL-1*β* or IL-18 [63, 76, 77] where mitochondrial ROS activate the inflammasome, which then induces the maturation and secretion of these proteins (ROS: reactive oxygen species; STX:, syntaxin; IL: interleukin; MIF: macrophage migration inhibitor factor; Acb1: acyl coenzyme A-binding protein).

The autophagic pathway has recently been related to the secretion of proteins from different cells. In this regard, many proteins known to be secreted by an unconventional route are known to be regulated by autophagy or their release is affected by knockdown of ATG proteins. Here, we review the proteins whose secretion has been shown to be regulated by autophagy (Figure 2, Table 1) and we later discuss the implications of the modulation of autophagy in protein secretion for cancer progression and treatment. Importantly, the term “secretory autophagy” is used to describe the process in which the canonical autophagic pathway takes part in the secretion of proteins by transporting them in the autophagosome directly to the plasma membrane, to MVB (multivesicular bodies), or to secretory lysosomes for their extracellular release. Thus, instead of inducing autophagosomal cargo degradation, secretory autophagy leads to the expulsion of the autophagosomal content to the extracellular space and it has a positive effect on protein secretion, since inhibition of autophagy reduces protein secretion (Table 1). This pathway would need specific cargo receptors as well as specific SNARE vesicular fusion proteins. On the other hand, another pathway has been described in which inhibition of autophagy leads to changes in protein secretion, particularly increased cytokine production in immune cells. In this case, autophagy has a negative effect on protein secretion since inhibition of autophagy increases protein secretion (Table 1), and this effect has been proposed to be mediated by increased mitochondrial reactive oxygen species (ROS) caused by decreased mitophagy. In the following sections, we discuss the proteins whose secretion is known to be modulated by the autophagic pathway, either because they are released through secretory autophagy or because inhibition of autophagy regulates their secretion, since both pathways would be affected by the modulation of autophagy for cancer therapy.

## 6. Secretory Autophagy

One of the first evidences indicating that autophagy was involved in the secretion of proteins came from studies in a mouse model of Chrohn's disease, a complex inflammatory disease of the intestine in which *ATG16L1* is one of many known risk alleles in patients [61]. So, in intestinal hypomorphic *ATG16L1* and intestinal *Atg5^−/−^* mice, autophagy deficiency mostly affected Paneth cells within the intestinal epithelium. These cells, whose normal function is to secrete both lysozyme and antimicrobial peptides, presented disorganized or diminished lysozyme-containing granules and increased lysozyme diffuse intracytoplasmic staining [61]. Thus, the process of autophagy was shown to have an important role in the maintenance of the granule exocytosis pathway in Paneth cells. More recently, lysozyme was found to be localized to autophagosomes (double-membrane, LC3^+^/p62^−^ vesicles) of *S. typhimurium*-infected Paneth cells. These autophagosomes were not targeted for lysosomal degradation but accumulated at the apical surface of Paneth cells for lysozyme secretion, indicating an important role for autophagy in the secretion of this antimicrobial protein [62]. In this work, lysozyme secretion was impaired in the intestinal crypts of *S. typhimurium*-infected mice treated with 3MA or in mice mutant for *Atg16L1^T300A^*, which impaired autophagy, but not by chloroquine treatment, indicating an important role for the initial steps of the autophagic pathway but not the degradation step of autophagy in the secretion of this protein. Secretory autophagy was induced by ER stress and was dependent on Myd88, a toll-like receptor (TLR) adaptor but specifically on dendritic cells. Treatment of *Myd88 ^−/−^* mice with recombinant IL-22 restored secretory autophagy of lysozyme in Paneth cells, indicating that Paneth cell secretory autophagy requires activation of dendritic cells to allow secretion upon ER stress in Paneth cells. Since Paneth cells are specialized intestinal cells that secrete antimicrobial proteins, including lysozyme, and since pathogenic microbes can trigger ER (endoplasmic reticulum) stress that interferes with protein secretion, the authors suggest that during *S. typhimurium* infection, autophagy is induced in Paneth cells where the secretion of lysozyme is rerouted to an alternative secretion pathway which involves the transport of lysozyme inside a specialized secretory autophagosome which is not targeted for degradation (since it was negative for p62 which targets proteins to be degraded by autophagy), preserving the antimicrobial function of Paneth cells [62].

More mechanistic studies have been made on the role of secretory autophagy in the release of IL-1*β* from mammalian cells. This proinflammatory cytokine lacks an ER-localization peptide, accumulates in the cytosol in its inactive form, and is later activated by caspase-1 cleavage for secretion by an unconventional route which involves inflammasome activation and autophagy [63–65]. So, the induction of autophagy by starvation in response to conventional NLRP3 inflammasome agonists has been shown to lead to enhanced IL-1*β* secretion in LPS-stimulated macrophages [64] and autophagy-mediated secretion was dependent on the inflammasome components ASC and NLRP3. In agreement with the previous observation, other inflammasome-dependent cytokines, like IL-18, also showed enhanced secretion after autophagy induction [64]. Importantly, in the same study, IL-1*β* was found to colocalize with Rab8a and LC3 and IL-1*β* secretion was decreased by Cre-mediated excision of *Atg5*, by lysosomal inhibition of autophagy with bafilomycin A or by Rab8a (a regulator of polarized sorting to plasma membrane) or GRASP55 (Golgi-associated protein required for unconventional secretion) knockdown. Also, cathepsin B was found to be secreted along with the inflammasome substrates. The mentioned evidence suggests a model in which autophagosomes have a direct role in the delivery of inflammasome-activated proteins to the plasma membrane and indicates a positive role for cathepsin B in IL-1*β* activation and extracellular delivery by autophagy.

Importantly, specialized secretory autophagosomes involved in the secretion of IL-1*β* or ferritin have already been identified [66]. In this work, upon lysosomal damage, TRIM16, together with galectin-8, acted as a receptor for IL-1*β* targeting it to LC3II-positive autophagosomes. Fusion with the plasma membrane was dependent on Sec22b on the autophagosome and on SNAP23/29 and STX 3/4 on the plasma membrane. Importantly, the secretion of IL-1*β* was STX17 (a SNARE involved in the fusion with the lysosome) independent, suggesting that secretory autophagy utilizes specialized “secretory” autophagosomes that would eventually fuse with the plasma membrane and that avoid cargo degradation in the lysosomes [66].

In yeast cells, another protein has been identified whose secretion depends on autophagy [67, 68]. An acyl coenzyme A-binding protein, Acb1, is a secreted protein lacking an ER-localization sequence involved in yeast sporulation in response to nitrogen starvation. Acb1 secretion was found to be independent of the conventional secretory pathway, dependent on the presence of *ATG* genes and proteins, on Grh1 (GRASP), and was also induced by rapamycin treatment [67, 68]. Interestingly, Acb1 secretion did not require fusion with the vacuole and required components of the multivesicular body endosomal compartment, indicating that Acb1-containing autophagosomes bypass the fusion and instead they fuse with endosomes or MVBs en route to the plasma membrane [68]. Yeast mutants which failed to secrete Acb1 showed similar levels of intracellular Acb1 protein and were deficient in its secretion but not in its processing, indicating that the pathway described was a protein secretion and not a degradation pathway [67].

Autophagy-mediated secretion has also been linked to major neurodegenerative diseases. In Parkinson's disease (PD), where both the proteasome and autophagy have been involved in the degradation of *α*-synuclein aggregates, autophagy has also been linked to the secretion of *α*-synuclein, indicating its potential role for interneuronal transmission of *α*-synuclein and PD [69, 70]. In this regard, in a PD model involving overexpression of an aggregation-prone *α*-synuclein and of TPPP/p25a, a microtubule-binding protein involved in *α*-synuclein-aggregate formation, *α*-synuclein was localized to autophagosomes since it colocalized with autophagy markers LC3 and p62/SQSTM1, but these autophagosomes did not fuse with lysosomes. This study showed that TPPP/p25a impaired autophagic flux at the lysosomal fusion level and induced *α*-synuclein secretion, similarly to autophagic-flux inhibitor treatment. Importantly, *α*-synuclein secretion was decreased by *ATG5* knockdown [69]. In a similar study, in different PD models of neurons overexpressing *α*-synuclein, lysosomal inhibition increased *α*-synuclein secretion and its localization to LC3II- and p62/SQSTM1-positive extracellular vesicles [70]. Other proteins found in extracellular vesicles from bafilomycin-treated neurons were VPS35, ATP6V1A, and LAMP2 [70]. Both studies suggest an important role for autophagosome formation and autophagosome fusion with the lysosomes in the regulation of extracellular vesicle secretion. Thus, while autophagosome formation could directly deliver contents to the multivesicular body as well as to the lysosomes, autophagic flux inhibition with lysosomal inhibitors could promote enhanced delivery of autophagosomal material to vesicles and their extracellular release.

Autophagy has also been closely related to Alzheimer's disease (AD). AD brain pathology involves the formation of intracellular amyloid beta (A*β*) peptide and tau protein aggregates as well as extracellular A*β* plaques [71, 72]. Impaired autophagic flux has been described in neurons of AD mouse models, and autophagosomes have been related to the generation of the A*β* peptide [71]. In agreement with impaired autophagic flux in advanced AD, induction of autophagy by rapamycin lowered intracellular A*β* accumulation and extracellular plaque load and prevented learning and memory deficits in a mouse model of AD but only when administered prophylactically and not in mice with established plaques and tangles [73]. Moreover, amyloid precursor protein transgenic mice with conditional knockout of *Atg7* in the forebrain excitatory neurons drastically accumulated intracellular A*β* and presented reduced extracellular A*β* plaque formation due to impaired secretion of A*β* [72]. Altogether, these findings underscore the importance of autophagy for the maintenance of neuronal homeostasis but could promote AD pathology by promoting A*β* extracellular plaque formation.

Several studies have also linked the autophagic pathway to the release of secretory lysosomes in a physiological setting. For instance, autophagy-related proteins have been shown to mediate osteoclast ruffled border formation and their secretory function by directing secretory lysosomes to the plasma membrane for fusion and secretion of cathepsin K [74]. Also, secretory granules of mast cells have been found to be LC3II^+^ and CD63^+^ (a marker of secretory lysosomes) and autophagy was found to have a crucial role in mast cell degranulation and the release of histamine and *β*-hexosaminidase [75].

In conclusion, secretory autophagy involves the formation of a specialized autophagosome (LC3II^+^, double-membrane structure) which sequesters cytoplasmic cargo for secretion instead of degradation. A precise understanding of how secretory lysosomes bypass fusion with the lysosome to avoid degradation remains to be described. The discovery of specialized receptors and fusion proteins that mediate secretion which permit modulation of this secretory pathway is likely to have implications in a pathological setting.

## 7. Enhanced Protein Secretion Caused by the Inhibition of Autophagy

In contrast to the previous studies where autophagy induction leads to enhanced secretion of proteins, other studies have reported the opposite: pharmacological or genetic inhibition of autophagy caused an increase in protein secretion of diverse proteins, particularly proinflammatory cytokines. Of particular interest is the case of IL-1*β* since we have previously mentioned studies in which autophagy induction by starvation in response to conventional NLRP3 inflammasome agonists increased IL-1*β* secretion in LPS-activated macrophages [64, 65]. In this regard, the opposite effect has also been described: enhanced IL-1*β* secretion has also been described after inhibition of autophagy, also in LPS stimulated macrophages. The first report linking the autophagic pathway to the secretion of IL-1*β* came from Saitoh et al. in 2008 [63]. In this study, the authors found that Atg16L1-deficient macrophages showed increased secretion of IL-1*β* but not of other proinflammatory proteins (IL-6, TNF*α*, and IFN*β*) to the culture medium upon LPS stimulation [63]. In this study, Atg16L1 deficiency caused accumulation of ROS after LPS exposure as well as caspase-1 activation and IL-1*β* cleavage [63]. Although the precise mechanism by which the production of ROS induced the activation of the inflammasome was not fully described in this work, a different group also described increased IL-1*β* and IL-18 but not TNF secretion after inhibition of autophagy with knockout of *Map 1lc3b* or *Becn1* in LPS-activated macrophages [76]. In this work, Nakahira et al. showed that inflammasome activation induced by autophagy inhibition in LPS-treated macrophages was dependent on the presence of increased mitochondrial ROS, decreased mitochondrial membrane potential, and mtDNA (mitochondrial DNA) release to the cytosol [76]. The authors also showed that mitochondrial ROS activated the NLRP3 inflammasome, and this activation was necessary for mtDNA release to the cytoplasm since it does not occur in NLRP3-deficient macrophages. Once in the cytoplasm, mtDNA activated the AIM2 inflammasome, which induced the secretion of IL-1*β* and IL-18 [76]. In agreement with the previous observations, Harris et al. described ROS-dependent IL-1*β* secretion after pharmacological inhibition of autophagy with 3MA or *beclin1* knockdown in LPS-activated macrophages [77]. Importantly, pharmacological autophagy inhibition with 3MA did not affect IL-6, IL-18, or TNF*α* secretion. The authors observed colocalization of IL-1*β* with GFP-LC3-stained autophagosomes which they interpret as pro-IL-1*β* being degraded by autophagosomes. In the same work, the authors showed that rapamycin treatment decreased IL-1*β* secretion in LPS-injected mice, indicating that not only the inhibition of autophagy induced the secretion of IL-1*β* but that its induction decreased it [77].

In this regard, oxidized mtDNA has been shown to be an important activator o the NLRP3 inflammasome [65]. The NLRP3 inflammasome is a sensor of specific pathogen, host, and environmental danger molecules which requires an initial priming signal, usually induced by TLR stimulation, required for the transcriptional induction of NLRP3 and pro-IL-1*β*. Upon priming, stimulation of a functional NLRP3 can be induced by a series of triggers [78]. Regarding LPS-induced IL-1*β* secretion induced by the inhibition of autophagy, mtDNA oxidation induced by the accumulation of damaged mitochondria due to decreased mitophagy, could be the second signal for inflammasome activation and increased IL-1*β* secretion. Although both works describing the role of autophagy in IL-1*β* secretion seem contradictory, it is important to mention that in the first case [64], Dupont et al. used conventional inflammasome agonists as nigericin to activate the inflammasome, while in the second case [63], Saitoh et al. used autophagy inhibition as the second signal for inflammasome activation. The authors also proposed that differences could be due to inhibition of basal versus starvation or mTOR inhibitor-induced autophagy [64, 79].

More recently, a similar mechanism in which inhibition of autophagy increased the secretion of a proinflammatory cytokine has been described for macrophage migration inhibitory factor (MIF) from LPS-activated macrophages. In this work, inhibition of autophagy with 3MA, *Atg5* siRNA, or *Atg7* knockout increased MIF secretion to the culture medium. This secretion occurred together with an increase in mitochondrial ROS and could be decreased with antioxidants [80]. The importance of the anti-inflammatory role of autophagy has been demonstrated *in vivo*, since Atg16L1 deficiency increased the production of IL-1*β* and IL-18 in a model of chemically induced colitis in mice [63] and in mouse models of sepsis where lack of autophagy caused more susceptibility to endotoxemia with increased IL-1*β* and IL-18 serum levels [76].

## 8. Autophagy and Its Interactions with the Vesicular Trafficking System

Autophagy interacts at different levels with the endolysosomal as well as with the exosome biogenesis and secretion machinery both in normal and cancer cells [81, 82]. Degradative autophagosomes can merge with the MVB to give rise to amphisomes, which later fuse with lysosomes for their degradation. This fusion depends on RAB11 [83], while RAB27a has been associated with fusion of the MVB to the plasma membrane [81]. Also, since fusion of MVBs with the plasma membrane results in the extracellular release of exosomes, induction of autophagy by starvation has been shown to decrease exosome secretion by diverting MVBs to the autophagic-lysosomal pathway for their degradation [83].

Different mechanisms of autophagy (macroautophagy and microautophagy) have been suggested to have an important role in cargo delivery to vesicles of the endosomal/exosomal system. Inhibition of autophagy has been shown to decrease the amount of cytosolic proteins in late endosomes, which are components of the MVB which can be targeted for degradation or released as exosomes. On the other hand, cytosolic proteins like GAPDH have been found to be secreted in exosomes even in the absence of autophagy, indicating that macroautophagy only partially contributes to the delivery of cytoplasmic proteins to late endosomes and that in the absence of autophagy, cargo proteins can be transported by a different pathway [84].

Thus, it has been suggested that a specialized form of autophagy has the main role in exosome cargo loading. Selective incorporation of proteins during exosome biogenesis and the mechanisms of invagination occurring during maturation of the MVB have been proposed to involve a type of endosomal microautophagy [84, 85]. Microautophagy is a type of autophagy characterized in yeast which involves direct internalization of cytosolic cargo through invaginations of the lysosomal membrane [84]. Thus, a specialized type of microautophagy, endosomal microautophagy, occurring in late endosomal MVBs has been proposed to be responsible for the delivery of cytosolic proteins to the vesicles. This process was shown to be mediated by the chaperone hsc70 and the ESCRT systems [84]. This endosomal microautophagy is a process by which autophagy contributes to the secretion of cytosolic proteins but seems to be different from secretory autophagy since it involves direct delivery of cytosolic proteins to late endosomes and is independent of ATG proteins, which participate in macroautophagy but not in microautophagy. On the other hand, delivery of proteins to the MVB during secretory autophagy requires their transport in the autophagosome and a direct interaction with the MVB as has been shown for Acb1 [67, 68], IL-1*β* [64], *α*-synuclein [69, 70], and annexin A2 [86].

Exosomes are characterized by the presence of proteins involved in their biogenesis such as Alix, TSG101, HSP70, and tetraspanins as well as cell type-specific proteins, DNA, RNA, and lipids [81]. In this regard, an important interaction of the autophagic machinery with Alix, an ESCRT associated protein, has recently been described [87]. ATG12 and ATG3 are both core autophagy components, and their conjugation (ATG12-ATG3) has been shown to be necessary for basal but not starvation-induced autophagy. This interaction is also necessary for late endosomal to lysosome trafficking and for lysosome biogenesis [87].

## 9. Autophagy-Mediated Secretion in Cancer

Secreted proteins are known to play important roles in supporting the hallmarks of cancer [88]. In this regard, autocrine or paracrine signaling in cancer cells is known to sustain excessive proliferation, reduced apoptosis, immune cell regulation, angiogenesis, alterations in energy metabolism, and development of resistance against cancer therapy [3, 59].

In cancer, the regulation of autophagy has been shown to have important effects on protein secretion. Perhaps the first evidence that autophagy could regulate secretion in a cancer-related setting came from a study in oncogene- (Ras-) induced senescence in human fibroblasts [89]. Cellular senescence is a state of stable cell cycle arrest which can work as a failsafe program in response to a variety of insults during transformation. In this work, autophagy was activated during senescence, and it was responsible for senescence-associated secretion of IL-6 and IL-8 through a posttranslational mechanism, since the mRNA levels of IL-6 and 8 were higher in *Atg 5/7* knockdown cells [89]. Mechanistically, it was proposed that during oncogene-induced senescence, the rough endoplasmic reticulum and autophagic vacuoles colocalized with mTOR at the trans-Golgi network in an area termed the TOR-autophagy spatial coupling compartment, TASCC [90]. Localization of mTOR to this complex was responsible for driving the synthesis of IL-6/8. In this work, amino acid depletion or dominant negative expression of Rab-GTPases decreased mTOR recruitment to the TASCC. The authors proposed that during oncogene-induced senescence, spatial coupling of the cells' catabolic (autophagic vacuoles) with the anabolic (mTOR, ER, Golgi) machinery augments their respective function and facilitates mass synthesis of secretory proteins like IL-6/8 [90]. Importantly, TASCC formation was dependent on brefeldin A [90], which blocks ER to Golgi protein transport, indicating the need for a functional conventional pathway for this secretory phenotype.

In a similar work, Lock et al. [57] described autophagy-mediated secretion of protumorigenic factors in a RAS-driven model of invasive breast cancer. In this study, autophagy was necessary for invasiveness and epithelial-to-mesenchymal transition in RAS-transformed MCF10A breast cancer cells and was also necessary for the secretion of proinvasive factors like IL-6, matrix-metalloproteinases 2 and 9, and WNT5A [57]. Also, in agreement with the proinflammatory role of autophagy, a recent work has also described autophagy-dependent inflammation (increased secretion of CSF3/G-CSF, CXCL1, IL-6, TREM1, CCL2, CCL3/MIP-1*α*, IL-1*β*, and CXCL2) in response to UVB radiation prior to tumorigenesis. Secretion of these cytokines from UVB-irradiated mice was blocked by conditional *Atg7* KO in the skin [91].

In contrast, although most of the evidence shows that autophagy is necessary for the secretion of proinflammatory cytokines like IL-6, there is also evidence showing that the inhibition of autophagy by knockdown of *ATG* genes decreased IL-6 secretion in autophagy-dependent breast cancer cell lines but increased its secretion in autophagy-independent cells [40]. This was related to the maintenance of cancer stem cells since IL-6 supplementation increased mammosphere formation in *ATG7* shRNA-expressing cells and was associated to dependence on autophagy for survival [45]. Thus, whether autophagy serves a proinflammatory or anti-inflammatory function seems to be context- and cell type-dependent.

Regarding the anti-inflammatory role of autophagy, in a mouse model of breast cancer, Wei et al. [56] found that suppression of autophagy by *FIP200*^−/−^ decreased mammary tumor initiation and progression. Decreased tumorigenesis occurred together with elevated production of chemokines in tumor cells and increased IFN*γ*-producing CD8^+^ and CD4^+^ (Th1) T lymphocytes in the tumor microenvironment [56]. In the same study, *FIP200*^−/−^, CD8^+^ T cell-depleted animals developed mammary tumors with a similar kinetics as the autophagy-competent control mice, indicating that decreased tumorigenesis in *FIP200*^−/−^ mice was due to increased chemokine secretion and the promotion of an antitumoral immune response.

Other studies have linked the inhibition of autophagy with increased secretion of cytokines from tumor cells. In this regard, in a *Kras*-driven non-small cell lung cancer (NSCLC) mouse model with a concurrent deletion of *Atg7* to inhibit autophagy in the tumors, the authors found a decrease in tumor growth with accumulation of defective mitochondria. Importantly, *Kras*-driven tumors, which normally formed adenomas and carcinomas, diverted to more benign oncocytomas in the absence of A*tg7*, indicating that the functional status of autophagy determines the tumor fate [51]. Despite decreased tumor burden, mice with *Atg7*-null tumors died from pneumonia with an increased inflammatory response. Interestingly, increased overall survival in the same model was observed only when *p53* was deleted together with *Atg7* as these mice did not show extensive inflammatory responses [51]. Thus, specific mutations present in the tumor might determine the role of autophagy inhibition on tumor cell-induced inflammation. This will be an important element to be considered when manipulating autophagy, since p53 is the most frequent tumor suppressor gene mutated in human cancers with diverse and context-dependent effects on cellular function [92].

Another protein whose secretion has been shown to be regulated by autophagy is HMGB1 [64, 93] (high-mobility group B1 immune modulator protein). Of note, HMGB1 is a nuclear protein which is not secreted in normal conditions and does not need to be processed by the inflammasome [64], indicating that the autophagic process modulates secretion by regulating different cellular pathways. HMGB1 is an immunogenic stimulator that is normally present in the cell nucleus and is considered to be released together with other alarmins during necrotic cell death upon plasma membrane rupture [94]. In cancer cells undergoing cell death induced by a targeted toxin, knockdown of *ATG* proteins prevented HMGB1 release [93]. These findings indicate that the levels of autophagy in a dying cell might determine the immunogenicity of this process at least partly by regulating the secretion of HMGB1 [93]. Another alarmin whose secretion has been proposed to be regulated by autophagy is ATP [47]. In this regard, autophagy-competent cancer cells treated with immunogenic chemotherapy, induced ATP secretion and a therapeutic immune response and this effect was not observed in autophagy-deficient (*Atg5* or Atg7 knockdown) cancer cells [47].

In contrast to the above-mentioned studies which suggest that, at least in cancer therapies with immunogenic potential, cell death with autophagy could promote a better long-term therapeutic response, emerging evidence suggests that in a different setting, autophagy could have an important role in the inhibition of the antitumor immune response. In this regard, hypoxia, an imbalance between increased oxygen consumption by tumor cells and an inadequate oxygen supply caused by cancer cell proliferation and defective tumor vascularization, has been shown to be an important regulator of tumor cell adaptation to low-oxygen conditions that can reshape tumors as well as their microenvironment [95]. These responses are known to be mediated by hypoxia-inducible factors (HIFs), transcription factors that mediate gene expression networks related to characteristics of malignancy, including the induction of autophagy [95]. Hypoxia-induced autophagy has been related to resistance to therapy [96] and avoidance of immune destruction [97]. Regarding the latter, it has been shown that HIF-1*α* can induce PD-L1 (programmed cell death ligand-1) expression to avoid cytolytic T lymphocyte (CTL) recognition [95] as well as BNIP3/BNIP3L, which induces autophagy that has been related to the development of resistance to CTL-mediated lysis. In this regard, pharmacological or genetic inhibition of hypoxia-induced autophagy decreased STAT3 phosphorylation in hypoxic tumor cells and restored tumor cell susceptibility to CTL-mediated lysis [97]. Although this work does not explore the relationship of secretion regulated by autophagy in resistance to cell lysis, cytokine secretion is likely to have a role in this phenotype since STAT3 is known to have an important role in the regulation of inflammation [98].

Despite the possible relationship of autophagy with the antitumoral immune response that we have previously discussed, a recent work found no changes in antitumor adaptive immunity in mouse models of melanoma and breast cancer after autophagy inhibition with *Atg* gene knockdown or with chloroquine/hydroxychloroquine treatment [99]. Thus, the precise role of autophagy in mediating the immunogenicity of tumor cells remains to be established.

Finally, despite controversial results in the literature and the context-dependent role of autophagy on protein secretion, the importance of identifying secreted proteins regulated by autophagy was evidenced in a recent work in melanoma [100]. In this work, melanoma tumor cells with low autophagy had a different secretome than their high-autophagy metastatic derivatives. High-autophagy melanoma cell lines presented higher levels of IL-1*β*, CXCL8, LIF, FAM3C, and DKK3 with known roles in inflammation and tumorigenesis. Levels of these proteins increased after autophagy induction and decreased with *ATG7* silencing in high autophagy cells. The authors found high levels of autophagy-regulated secreted proteins in serum of patients with high autophagy and suggest that serum levels of these proteins could be used as markers of autophagy levels in tumor cells which could be targeted with autophagy inhibitors [100].

## 10. Discussion

Evidence suggests that whether autophagy serves an anti-inflammatory or inflammatory role in cancer seems to depend on the stage of tumorigenesis, on the cancer type, and on the secreted factor being studied. Importantly, autophagy has been related to the secretion of proteins whose release is regulated by both conventional and unconventional pathways, and autophagosomes are also closely linked with the endosomal-vesicular pathway, indicating that it could be playing diverse or even opposing roles on protein secretion depending on the cellular context.

Indeed, autophagy has an important role in the regulation of protein secretion in several types of cells. Mechanistically, two major autophagy-mediated secretion pathways have been described. The first one, secretory autophagy [13], involves a halted autophagic flux in which autophagosomes do not fuse with the lysosome and cargo-containing autophagosomes are directed to the plasma membrane or to multivesicular bodies for secretion, as has been described for Acb1 [67, 68], lysozyme [62], IL-1*β* [64, 66], and *α*-synuclein [69, 70]. Important mediators of this pathway are proteins necessary for plasma membrane fusion like Rab8*α* [64, 69], Sec22b, SNAP23/29, and STX3/4 [66]; absence of STX17 [66], which is necessary for fusion with the lysosome and GRASP proteins [64, 67]; and possibly peroxisomal signaling [61, 67] (Figure 2). In the second pathway, autophagy seems to serve as an antioxidant mechanism by decreasing damaged mitochondria (Figure 2). In this case, inhibition of autophagy would increase mitochondria which would otherwise be degraded. Cytoplasmic mtDNA leaking from mitochondria with low intermembrane potential could then activate the inflammasome as has been proposed for proinflammatory cytokine secretion like IL-1*β*, IL-18 [63, 76, 77], or MIF [80]. If there is a specific autophagy-regulated pathway for each one of the secreted proteins described, if specific markers for the vesicles involved exist, or if there is a combination of both pathways mentioned, as has been proposed for IL-1*β* [64], remains to be determined.

It will thus be important to establish how autophagy regulates secretion from cancer cells, if this regulation is similar to the one observed in non-transformed cells, in what cancer types or cancer stage autophagy is regulating secretion, and if protumorigenic or immune-regulating factors are being modulated by autophagy to better target autophagy for the treatment of cancer. Importantly, many of the proinflammatory cytokines regulated by autophagy in immune cells have not been studied in models of autophagy inhibition in the context of cancer. In this regard, IL-1*β* has been shown to induce epithelial to mesenchymal transition in breast cancer cells [101] and IL-1 signaling has been related to inflammation and aggressiveness due to the modulation of antitumor immunity in the same type of cancer [102]. Also, MIF, whose secretion has been shown to be increased after inhibition of autophagy [80], has been found to be elevated in different types of human cancers and is known to promote tumorigenesis through stimulation of proliferation, angiogenesis, metastasis, and inhibition of the antitumoral immune response [103]. This will be an important factor to evaluate in clinical trials currently using autophagy inhibition for the treatment of several types of cancer, particularly in those types of cancer where the antitumoral immune response has an important role in patient response.

Importantly, some of the proteins that have been identified as being regulated by autophagy in cancer, e.g., IL-6 and 8, are secreted by a conventional protein secretion route and their secretion is closely related to their transcription, underscoring the importance of understanding the relationship of the autophagic pathway to conventional protein secretion routes as well as to the regulation of their transcription factors like NF-*κ*B or STAT3, to establish how manipulation of autophagy during cancer therapy might affect the tumor microenvironment. In this regard both, IL-6 and 8 have been shown to have important roles in maintaining oncogenic signaling in cancer cells, in promoting cancer stem cell maintenance [104–106] and in the regulation of the tumor microenvironment [107]. Since autophagy inhibition has been shown to decrease IL-6 and 8 secretion, inhibition of autophagy during cancer therapy would decrease their secretion in cancer cells. However, increased IL-6 secretion has also been reported for some cancer cells [40], particularly those that are not dependent on autophagy for survival. This is an important consequence that needs to be addressed in clinical trials manipulating autophagy in those types of cancer where autophagy has not proven to be important for cancer cell survival. In these cases, autophagy inhibition could possibly induce cytokine secretion and promotion of tumorigenesis as well as escape from the antitumoral immune response.

Finally, since intercellular communication is an important feature of tumor aggressiveness and tumor cell-derived extracellular vesicles transmit oncogenic signals to the neighboring tumor cells or to the cells in the tumor microenvironment, it will be important to understand how the modulation of autophagy affects exosomal content or exosomal release from tumor cells or from the tumor microenvironment since it is likely that at least some of the effects observed by the modulation of autophagy during cancer therapy, especially in immune-competent animals, will be mediated by extracellular vesicle release.

It is probable that secretion induced by the modulation of autophagy during cancer therapy will have different and context- or tissue-dependent roles, just as the manipulation of autophagy for cancer therapy or the regulation of the antitumoral immune response. Nevertheless, since some of the consequences of the inhibition of autophagy could promote malignancy or have other undesirable consequences, it will be important to understand how autophagy modulates secretion and how manipulation of autophagy will affect secretion in order to effectively modulate autophagy and its effects on secretion for the purpose of cancer therapy as well as for the treatment of other diseases.

## Figures and Tables

**Table 1:** Proteins whose secretion is known to be regulated by autophagy. The table shows proteins whose secretion has been shown to be regulated by alterations in the autophagic pathway, the methods used to manipulate autophagy, and the effect of autophagy on secretion: positive, if autophagy inhibition impairs secretion, or negative, if autophagy inhibition increased secretion (3MA: 3-methyl adenine; LPS: lipopolysaccharide; CQ: chloroquine; kd: knockdown; Baf: bafilomycin A1; EMT: epithelial to mesenchymal transition).

Secreted protein	Protein function	Method(s) used to modulate autophagy	Autophagy's effect on secretion	Ref.
Acb1	Acyl-CoA-binding protein involved in yeast sporulation	*ATG1*, *5*, *6*, *7*, *8*, *9*, *12*, *17* and *VAM7* mutant yeast; rapamycin	Positive; genetic inhibition of autophagy decreased and rapamycin increased secretion. Fusion of the autophagosome with the vacuole was not related to secretion.	[67, 68]
Amyloid-*β* peptide	Element of the amyloid plaques involved in Alzheimer's disease	*Atg7^−/−^*	Positive; genetic inhibition of autophagy caused intracellular Ab accumulation and reduced amyloid B peptide secretion.	[72]
Annexin A1	Regulator of the inflammatory process	*Beclin1* kd, 3MA, and *Atg5^−/−^*	Positive; genetic inhibition of autophagy or 3MA treatment decreased secretion induced by inflammasome activators. Found in screening experiments of secreted proteins regulated by autophagy.	[66, 70, 108]
Annexin A2	Ca^2+^-dependent phospholipid-binding protein	*ATG5* kd, 3MA, and lysosomal inhibitors	Positive; genetic inhibition of autophagy or 3MA treatment decreased secretion in IFN-*γ*-stimulated lung epithelial cells. Found in screening experiments of secreted proteins regulated by autophagy.	[70, 86]
*α*-Synuclein	Aggregation-prone protein involved in Parkinson's disease	*ATG5* kd, TPPP/p25 which impaired autophagic flux at the lysosomal fusion step, trehalose, and lysosomal inhibitors	Positive; autophagy inhibition in the presence of TPP/p25 decreased secretion. Autophagosome-lysosome fusion impairment was necessary for secretion, and autophagosome-lysosome fusion impairment enhanced secretion of an LC3/p62^+^ vesicle.	[69, 70]
*β*-Hexosaminidase	Lysosomal enzyme, indicator of mast cell degranulation	*Atg7^−/−^* and *Atg12* kd	Positive; genetic inhibition of autophagy decreased mast cell degranulation.	[75]
Cathepsin D	Lysosomal protease	*Beclin1* kd, 3MA, and *Atg5^−/−^*	Positive; genetic inhibition of autophagy or 3MA treatment decreased secretion induced by inflammasome activators. Found in screening experiments of secreted proteins regulated by autophagy.	[66, 108]
Cathepsin K	Bone resorption	*Atg5^−/−^*, *Atg7^−/−^*, and *Atg4^C74A^* dominant negative	Positive; autophagy inhibition decreased secretory lysosome delivery to the plasma membrane.	[74]
CXCL8	Chemokine produced by macrophages and epithelial cells	*ATG7* kd	Positive; autophagy inhibition decreased secretion.	[100]
DKK3	Glycoprotein with angiogenesis and invasiveness-promoting roles	*ATG7* kd	Positive; autophagy inhibition decreased secretion.	[100]
FAM3C	Secreted protein inducer of EMT	*ATG7* kd	Positive; autophagy inhibition decreased secretion.	[100]
Ferritin	Iron storage protein	*LC3B* kd	Positive; inhibition of autophagy decreased secretion in response to lysosomal damage.	[66]
Galectin 3	Lectin with affinity for *β*-galactoside glycoconjugates	*Beclin1* kd and 3MA	Positive; genetic inhibition of autophagy or 3MA treatment decreased secretion induced by inflammasome activators.	[108]
Histamine	Inflammatory response, component of mast cell granules	*Atg7−/−* and *Atg12* kd	Positive; genetic inhibition of autophagy decreased mast cell degranulation.	[75]
HMGB1	Alarmin normally present in the nucleus and released during cell death	*ATG5*, *7*, and *12* kd	Positive; genetic inhibition of autophagy decreased secretion in cancer cells treated with targeted therapy.	[93]
IL-1*β*	Inflammatory response	*Atg*5^−/−^, bafilomycin A [64], *beclin 1* kd, 3MA [108], *ATG16L1*, *LC3B* kd [66], and *Atg*7^−/−^ [91]	Positive; genetic [64] or pharmacological [108] inhibition of autophagy decreased secretion in response to inflammasome activation, lysosomal damage [66], or UVB irradiation [91].	[64, 66, 91, 108]
		Truncated *Atg16L1*, *Atg*7^−/−^, and 3MA [63, 77], *Map1lc3b*^−/−^ or *becn1*^−/−^ [76], and *becn1* kd [77]	Negative; genetic autophagy inhibition or PI3K inhibitor treatment induced secretion in LPS primed macrophages.	[63, 76, 77]
IL-6	Inflammation	*ATG5*, *ATG7*, *ATG12*, *beclin1* kd, and *Atg*7^−/−^	Positive; genetic inhibition of autophagy decreased secretion in cancer cell lines [40, 57, 89], in UVB irradiated skin [91], or in hepatitis virus infected hepatocytes [109].	[40, 57, 89, 91, 109]
		*ATG7* and *Beclin1* kd	Negative; genetic inhibition of autophagy increased secretion in a breast cancer cell line but not others.	[40]
IL-8	Chemotactic factor and neutrophil activator	*ATG5* and *ATG7* kd	Positive; genetic inhibition of autophagy decreased secretion in cancer cell lines [100] or in hepatitis virus-infected hepatocytes [109].	[100, 109]
IL-18	Proinflammatory cytokine	Truncated *ATG16L1* [63] and *Map1lc3b*^−/−^ or *Becn1*^−/−^ [76]	Negative; genetic autophagy inhibition induced secretion in mouse models of colitis or sepsis or in LPS-primed macrophages.	[63, 76]
		3MA or bafilomycin treatment	Positive; pharmacological inhibition of both initial and degradation phases of autophagy decreased secretion in allergen-induced IL-18 secretion.	[110]
LIF	Cytokine involved in hematopoietic differentiation, stem cell development, metabolism, and growth promotion	*ATG7* kd	Positive; autophagy inhibition decreased secretion.	[100]
Lysozyme	Antimicrobial protein	Hypomorphic *ATG16L1*, *Atg5^−/−^* [61] and *Atg16L1^T300A^*, 3MA, and CQ [62]	Positive; lysozyme secretion was impaired from Paneth cells by genetic inhibition of Atg genes or 3MA but not CQ treatment.	[61, 62]
Metalloproteinase 2/9	Extracellular matrix-degrading proteases	*ATG7* and *12* kd	Positive; genetic inhibition of autophagy decreased secretion.	[57]
MIF	Proinflammatory cytokine	*Atg5* kd, *atg7*^−/−^, and 3MA treatment	Negative; inhibition of autophagy increased MIF secretion in LPS-stimulated macrophages.	[80]
Neuropeptide Y	Neurotransmitter	*Atg16L1* kd	Positive; *Atg16L1* kd but not *Atg13* or *ULK1* kd decreased secretion in neuroendocrine cells.	[111]
